# Preventive effects of ^125^I seeds on benign restenosis following esophageal stent implantation in a dog model

**DOI:** 10.3892/mmr.2014.3130

**Published:** 2014-12-22

**Authors:** ZHEN GAN, JIAN JING, GUANGYU ZHU, YONGLIN QIN, GAOJUN TENG, JINHE GUO

**Affiliations:** Department of Intervention and Vascular Surgery, Zhongda Hospital, Southeast University, Nanjing, Jiangsu 210009, P.R. China

**Keywords:** iodine-125 seeds, fibroblasts, proliferation, esophageal stent, benign restenosis

## Abstract

The present study aimed to evaluate the effects of iodine-125 (^125^I) seeds on the proliferation of primary esophageal fibroblasts in dogs, and to assess the safety and preventive efficacy of ^125^I seed-pre-loaded esophageal stents in benign restenosis following implantation. Primary fibroblasts were cultured with various ^125^I seed activities, which were then evaluated using cell proliferation and apoptosis assays as well as cell cycle analysis using Annexin V/propidium iodide (PI) double staining and PI staining. Prior to sacrification, animals were submitted to esophageal radiography under digital subtraction angiography. Esophageal tissues were collected and examined for macroscopic, microscopic and pathological alterations. The results demonstrated a significant and dose-dependent inhibition of fibroblast proliferation and increased apoptosis following exposure to ^125^I seeds. G0/G1 fibroblast populations increased in a dose-dependent manner following treatment with ^125^I seeds, in contrast to cells in S phase. Four weeks following implantation, α-smooth muscle actin and proliferating cell nuclear antigen expression levels in the experimental group were significantly lower compared with those in the control group; in addition, eight weeks following implantation, esophageal inner diameters were increased in the experimental group. ^125^I seeds inhibited proliferation of dog esophageal fibroblasts via cell cycle arrest and apoptosis. In conclusion, ^125^I seed-pre-loaded esophageal stents inhibited benign hyperplasia in the upper edge of the stent to a certain extent, which relieved benign restenosis following implantation with a good safety profile.

## Introduction

Over the past two decades, esophageal stent placement has been considered a simple and effective method to relieve dysphagia in patients with advanced esophageal carcinoma (1,2). In addition, esophageal stents were shown to improve patients’ nutritional status and quality of life, resulting in an increased longevity (3–6). However, esophageal stents present obvious disadvantages (7); for instance, stent restenosis due to tissue hyperplasia on the upper edge of the stents constitutes a common long-term complication and presents major limitation for interventional therapies (8,9). Of note, a decrease in benign restenosis was reported, with a 22.2% incidence rate, following treatment with irradiated stents (10,11) compared with that of treatment with conventional stents, where benign restenosis incidence rates ranged from 30–60% (12,13). These high incidence rates can seriously affect the long-term efficacy of esophageal stents.

Numerous studies have shown that stricture generation following esophageal stenting was associated with fibroblast proliferation (14–16). Of note, it has been demonstrated that catheter-based intracoronary β and γ radiotherapy significantly altered the occurrence of subsequent restenosis, including the neointimal proliferation which occurs during the restenotic process (17–19). To the best of our knowledge, there are no existing studies demonstrating the incidence reduction of benign restenosis following esophageal stenting through inhibition of esophageal fibroblast proliferation. It is thought that these esophageal fibroblasts may deliver growth factors in conjunction with monocytes as the stimuli for restenosis (20).

However, fibroblasts may be inhibited using low-energy radiation of γ-rays produced by the widely clinically used iodine-125 (^125^I) seeds. The cytotoxicity of ^125^I seeds has been demonstrated in various cells derived from lung (21), colorectal (22) and prostate carcinomas (23). Therefore, ^125^I seed irradiation therapy was proposed for the treatment of brain tumors (24) and gastric cancer xenografts (25), among others.

In the present study, three clinically used ^125^I seeds with activities of 11.1, 22.2 and 33.3 MBq were evaluated for their inhibitory effects on esophageal fibroblast proliferation and their optimal doses were determined *in vitro*. Novel esophageal stents were designed using ^125^I seeds at the determined optimal inhibitory doses. ^125^I seed-preloaded stents were implanted in dogs in order to evaluate their preventive effects on benign restenosis as well as to perform safety studies. The present study aimed to provide a basis for the potential development of more effective and safer novel devices for clinical use in the treatment of benign restenosis.

## Materials and methods

### Animals

One-year-old male and female Beagle dogs (body weight, 11±0.1 kg) were provided by the Experimental Animal Center of Southeast University (Jiangsu, China). Beagle dogs were kept by the Experimental Animal Center of Southeast University under as 12-h light/dark cycle at 23°C and fed standard dog chow. All procedures and animal experiments were approved by the Animal Ethical Committee of the Southeast University and conducted in accordance with State and international regulations.

### Induction of dog esophageal fibroblasts

One Beagle dog was intravenously anesthetized with 1 ml/kg 3% sodium amobarbital (Sinopharm Chemical Reagent Co., Ltd, Shanghai, China) following one day of fasting. The dog then underwent layer by layer surgery and the esophagus was partly dissociated. Then, a 8×2 mm nitinol wire (Micro-tech, Jiangsu, China) was implanted into the esophageal muscle followed by incision sutures (layer by layer). Two weeks later, the esophagus was dissociated following the same procedure and the nitinol wire as well as the surrounding esophageal tissues were extracted.

### Primary culture of Beagle dog esophageal fibroblasts

Esophageal tissues were immerged in phosphate-buffered saline (PBS; Life Technologies, Grand Island, NY, USA) with high concentrations of penicillin-streptomycin (Biosharp, Seoul, Korea) for 10 min and washed 10–15 times in the same solution. Subsequently, the tissues were minced with ophthalmic scissors to ~1 mm^3^ and placed in culture medium. Minced tissues were then transferred to a centrifuge tube and digested with excess trypsin (HyClone Laboratories, Inc., Logan, UT, USA) at 37°C, with agitation every 5 min. The digestion was ceased when the medium became cloudy and free cells were observed under a microscope (BX53; Olympus, Tokyo, Japan). Throughout this experiment centrifugation was performed at 453 × g for 5 min and the supernatant was discarded. Low glucose Dulbecco’s modified Eagle’s medium (DMEM; HyClone Laboratories, Inc.) supplemented with 15% fetal calf serum (Hyclone Laboratories, Inc.), 100 U/ml penicillin (Biosharp) and 100 μg/ml streptomycin (Biosharp), were added to the resulting pellets. Cells were incubated in a humidified incubator (HERACELL 150i; Thermo Fisher Scientific, Waltham, MA, USA) with 5% CO_2_ at 37°C. The culture medium was removed 24–36 h following incubation and deformed adherent cells were observed under a microscope. Cells were washed with PBS and further incubated in 5 ml culture medium which was replaced every three days. Regular cell subcultures were performed for preservation at 85% confluency.

### Identification of Beagle dog esophageal fibroblasts

Detection of the relative expression of vimentin in fibroblasts was performed as previously described (26), using the Vimentin immunohistochemistry kit (Nanjing KeyGEN Biotech Co., Ltd, Jiangsu, China) according to the manufacturer’s instructions. Cells of passage three were seeded at a density of 3.0×10^5^ cells/ml into a six-well plate containing sterilized coverslips. Following two days of incubation, cells were fixed for 30 min with 4% paraformaldehyde (Sinopharm Chemical Reagent Co., Ltd), washed three times with PBS and then incubated in 3% hydrogen peroxide (Sinopharm Chemical Reagent Co., Ltd) at room temperature for 5 min. Following blocking with goat serum (Nanjing KeyGEN Biotech Co., Ltd) for 30 min, samples were incubated with mouse anti-human monoclonal anti-vimentin antibodies (1:100; Nanjing KeyGEN Biotech Co., Ltd) overnight at 4°C followed by incubation with biotin-labeled goat anti-mouse immunoglobulin G (1:100 in antibody diluents; Nanjing KeyGEN Biotech Co., Ltd) at 37°C for 30 min. Samples were then incubated with horseradish peroxidase-labeled streptavidin solutions (Nanjing KeyGEN Biotech Co., Ltd) at 37°C for 30 min and the signals were evaluated following the addition of diaminobenzidine (Nanjing KeyGEN Biotech Co., Ltd) and hematoxylin staining under an inverted microscope (BX53; Olympus).

### ^125^I seed types and development of an in vitro ^125^I seed irradiation model

**Figure 1:**
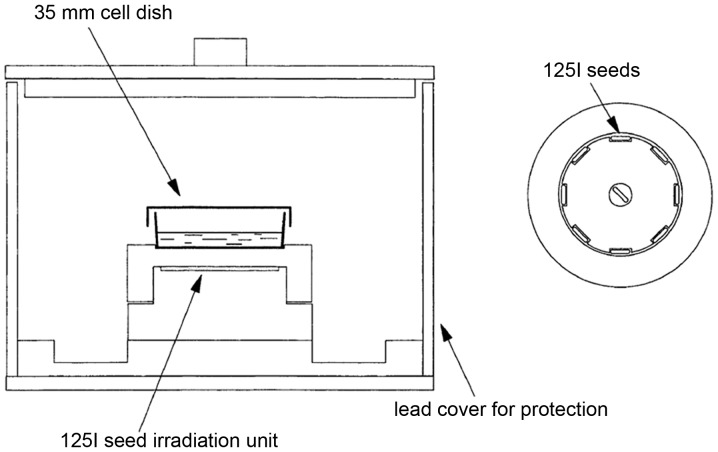
*In vitro* ^125^I seed irradiation model. (A) ^125^I seed irradiation model for cell experiments. The Petri dish was placed 6 mm above the irradiation unit and a 3-mm lead cover was constructed around the model for irradiation protection. (B) ^125^I seed irradiation unit. The unit consisted of a 35-mm diameter polystyrene panel with eight recesses equidistantly spaced at the circumference with one seed in the center.

In the present study, ^125^I seeds of clinical doses with 11.1, 22.2 and 33.3 MBq were purchased from GMS Pharmaceutical Co., Ltd. (Shanghai, China). The *in vitro* ^125^I seed irradiation model developed in the present study, as previously described (27), is shown in Fig. 1. One irradiation unit consisted of a 35-mm diameter polystyrene panel with eight recesses (4.5×0.8 mm) equidistantly spaced at the circumference with one seed in the center. When irradiated, one petri dish containing cells was placed 6 mm over the panel with a 3-mm lead cover around the outer surface for irradiation protection.

### ^125^I seed irradiation of cells

Fibroblasts were treated in the presence or absence of ^125^I seed irradiation with activities of 11.1, 22.2 and 33.3 MBq. Three replicates were used in the control group without irradiation, while nine replicates were evaluated in each experimental group. At passage three, 1.5×10^5^ cells/ml were seeded into 2 ml culture media, cultured overnight and subsequently incubated in irradiation units for 72 h. The average absorbed doses in cells within 72 h were 0.75, 1.50 and 2.25 Gy, respectively. These data were determined using a software provided by Beijing Tianhangkelin Technology Development Co, Ltd (Radioactive seed source implantation treatment planning system; China State Food and Drug Administration, approval no. 3700398 of 2009).

### MTT assay

Fibroblasts at a density of 1.5×10^5^ cells/ml in 200 μl were seeded into a 96-well plate. Following addition of 20 μl MTT solution (5 mg/ml; Beyotime Institute of Biotechnology, Jiangsu, China), plates were incubated in a humidified environment with 5% CO_2_ at 37°C for 4 h. Dimethyl sulfoxide (200 μl; Biosharp) was added to each well following supernatant removal to dissolve the purple crystals. Absorbance was then read at 490 nm on a microplate reader (Thermo Fisher Scientific) and the inhibition rate (IR) in each experimental group was calculated as follows: IR (%)=[(OD_control group_-OD_experimental group_)/OD_control group_]x100%.

### Assessment of cell apoptosis using Annexin V and propidium iodide (PI) double staining

Apoptosis was quantified using the Annexin V-Enhanced Green Fluorescent Protein Apoptosis Detection kit (KeyGEN Biotech Co., Ltd) according to the manufacturer’s instructions. In brief, 1 ml cells (1.5×10^5^/ml) were collected in flow cytometry tubes and resuspended in 500 μl binding buffer. Then 5 μl Annexin V and 5 μl PI were added to cell suspensions and the samples were incubated in the dark for 15 min at room temperature. Flow cytometry was performed using a FACSCalibur (BD Biosciences, San Jose, CA, USA). Data were acquired and analyzed using CellQuestPro software (BD Biosciences). Experiments were performed in triplicate. Annexin V and PI were set on the horizontal and vertical axes, respectively, and flow cytogram quadrants were divided as follows: Upper left, mechanically damaged cells; upper right, late apoptotic or necrotic cells; lower left, normal cells; and lower right, early apoptotic cells. Total apoptotic rate including early and late apoptotic rate were calculated.

### Assessment of cell cycle distribution using PI staining

Cells (1 ml; 1.5×10^5^/ml) were collected into flow cytometry tubes and fixed overnight in 1 ml ethanol (−20°C, 70%; Sinopharm Chemical Reagent Co., Ltd) at 4°C. Following centrifugation, supernatants were discarded and cells were resuspended in 0.5 ml PBS. Following addition of 1 ml DNA extraction buffer (Beyotime Institute of Biotechnology), the samples were incubated for 5 min at room temperature and subjected to centrifugation. The resulting pellets were resuspended and mixed thoroughly in 400 μl DNA dye solution (PI and RNAase; Beyotime Institute of Biotechnology) and stained in dark for 30 min. Samples were analyzed using flow cytometry as described above and data were acquired and analyzed using ModFitLT V2.0 software (Verity Software House, Topsham, ME, USA).

### Safety and efficacy evaluation of novel esophageal stents loaded with ^125^I seeds for prevention of benign restenosis in dogs

**Figure 2:**
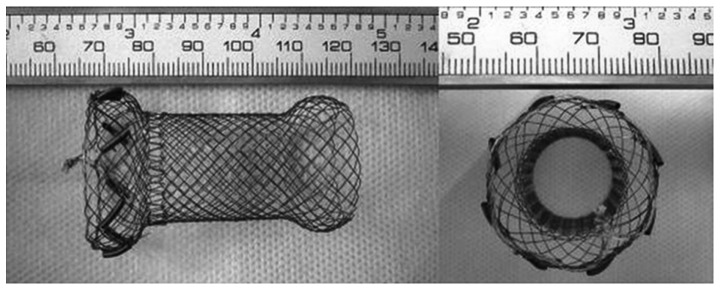
^125^I seed-loaded esophageal stent. Side view (left) and axial view (right) showing the eight equally-spaced ^125^I seeds on the upper edge.

The esophageal stents used in the present study were based on bare stents of 50 mm in length and 20 mm in diameter nickel-titanium alloy wires (Micro-tech) and eight sheathes made of alloy wires with a memory effect as holders for each ^125^I seed, equidistantly and symmetrically placed at the circumference of the upper edge of the stents (Fig. 2).

A total of 32 Beagle dogs were randomly divided into two groups of 16. In the experimental group, stents were loaded with eight ^125^I seeds in eight sheathes on the upper edge with the same activity of 33.3 MBq, as verified to be the optimum dose, and implanted into the esophagi of the dogs. In the control group, stents with empty seeds were used as implants. All dogs fasted for 12 h prior to surgery. Anesthesia was administered into forelimbs with intravenous injections of 1 ml/kg 3% sodium amobarbital. Dogs were then immobilized and neck incisions were made on the upper thorax. Dog esophageal walls were freed during the surgery and surrounded by polytetrafluoroethene (PTFE) membranes (MULTI-X9; Steriking, Bomlitz, Germany) with a predetermined size of 2×6 cm, which were used to fix the stents. The edges of the membranes were then sutured using no. 3 medical suture needles and surgical sites were sutured layer by layer. Esophageal stents were monitored using a digital subtracted angiography (DSA; Innova 3100; GE Healthcare, Little Chalfont, UK). Two days following stent implantation, dogs were intravenously rehydrated and one day later were fed with semi-fluid diets. The animals were regularly observed for general health conditions and dietary intake.

### DSA

At 1, 2, 4 and 8 weeks following implantation, four dogs per group were anaesthetized and esophageal radiography under DSA was performed using a Stenoscop 9000 small C-arm (Parameters: 70 kV, 150mA, 750×750 field; GE Healthcare) in order to detect loss and disclosure of ^125^I seeds as well as the displacement and patency of stents.

### Esophageal inner diameters

Animals were sacrificed using an injection of 10% KCl (Sinopharm Chemical Reagent Co., Ltd) into the forelimb vein. The stented esophageal segments were harvested along with adjacent tissues 20 mm above the stents, which were used for further evaluation. Morphological changes in the esophagus were assessed by measuring esophageal inner diameters which reflected the degree of restenosis.

### Hematoxylin-eosin (HE) staining and immunohistochemistry

The stented esophageal segments, along with adjacent tissues 20 mm above the stents, were harvested, fixed and paraffin (Specimen Model Factory, Shanghai, China) embedded. Sections were then stained with HE (Nanjing KeyGEN Biotech Co., Ltd) and immunohistochemistry (IHC) was performed in order to detect α-smooth muscle actin (SMA) and proliferating cell nuclear antigen (PCNA) expression, using mouse anti-human monoclonal anti-α-SMA antibodies (1:100 Beijing Biosynthesis Biotechnology Co., Ltd, Beijing, China), mouse anti-human monoclonal anti-PCNA antibodies (1:100; Beijing Biosynthesis Biotechnology Co., Ltd) and the PCNA IHC detection kit (Maxin, Fujian, China). Sections were incubated with the antibodies for 1 h at 37°C. Assays were performed following routine IHC procedures and the specimens were observed under a BX53 microscope (Olympus). Optical density values were determined using Image-Pro Plus 6.0 (MediaCybernetics, Rockville, MD, USA).

### Amino acid composition

Proteins were digested by acid hydrolysis as previously described (28). In brief, 80 mg tissue samples were placed into a 10-ml ampoule followed by the addition of 3 ml HCl (6 M; Sinopharm Chemical Reagent Co., Ltd). The ampoules were filled with nitrogen (Sinopharm Chemical Reagent Co., Ltd) and sealed. Following incubation at 110°C for 12 h, samples were cooled down and 0.28 M NaOH (Sinopharm Chemical Reagent Co., Ltd) was used for neutralization prior to analysis using an 8900 Automaticamino acid analyzer (Hitachi, Tokyo, Japan).

### Statistical analysis

All statistical analyses were conducted using SPSS version 18.0 (International Business Machines, Armonk, NY, USA). Values are presented as the mean ± standard deviation of three independent experiments performed in duplicate, unless otherwise stated. Statistical significance was evaluated using a Student’s t-test or one-way analysis of variance with a Dunnett’s test for post hoc analysis. P<0.05 was considered to indicate a statistically significant difference between values.

## Results

### Identification of Beagle dog esophageal fibroblasts

**Figure 3:**
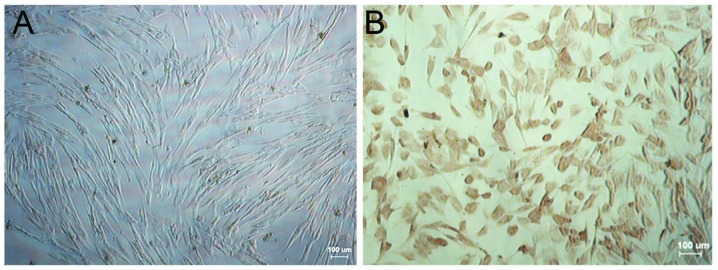
Identification of esophageal fibroblasts. (A) Esophageal fibroblasts at passage three were observed to be radial or spiral-shaped in growing areas as visualized under an inverted microscope. (B) Expression of vimentin in fibroblasts was determined using immunohistochemistry. A large number of brown-stained granules were observed in the cytoplasm (Scale bars, 100 μm).

The morphological and immunohistochemical characteristics of esophageal fibroblasts from Beagle dogs are shown in Fig. 3. Fibroblasts were translucent, spindle-shaped, elongated and radial or spiral-shaped following overgrowth (Fig. 3A). In addition, the relative specific expression of vimentin in fibroblasts was determined using immunohistochemistry, which revealed a large number of brown-stained granules in the cytoplasm (Fig. 3B).

### Effect of ^125^I seed irradiation on proliferation, cell cycle distribution and apoptosis in esophageal fibroblasts

For cell viability assays, absorbance was measured at 490 nm following exposure of primary fibroblasts to ^125^I seed irradiation *in vitro*. The IR in each experimental group was calculated as described above (Table I). The results demonstrated a significant dose-dependent inhibition of fibroblast proliferation (P<0.05), of which the most potent ^125^I seed activity was at 33.3 MBq with an IR of 45.33±2.59%.

**Figure 4:**
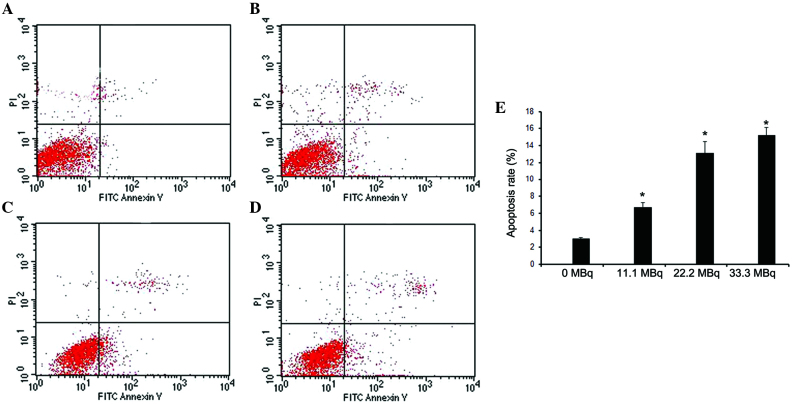
Effects of ^125^I seed irradiation on apoptosis in esophageal fibroblasts. Fibroblasts were treated with (A) 0, (B) 11.1, (C) 22.2 and (D) 33.3 MBq ^125^I seed irradiation. Apoptotic rates of fibroblasts were then determined using flow cytometry with Annexin V/PI staining. (E) Quantification of the apoptotic rates of fibroblast following irradiation treatment. Values are presented as the mean ± standard deviation. ^*^P<0.05 vs. 0 MBq. FITC, fluorescein isothiocyanate; PI, propidium iodide.

**Figure 5:**
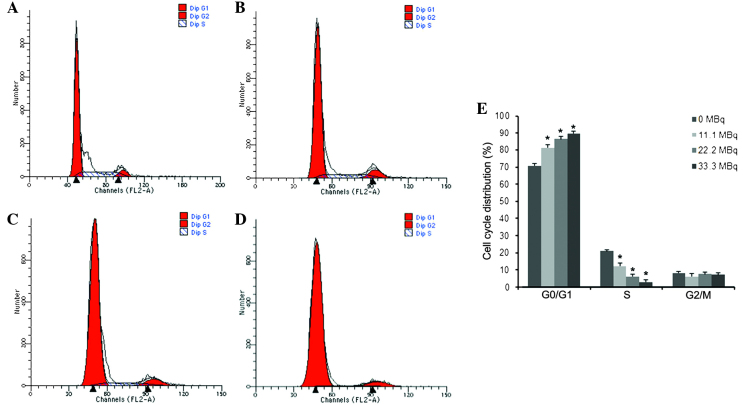
Effects of ^125^I seed irradiation on cell cycle distribution of esophageal fibroblasts. Fibroblasts were treated with (A) 0, (B) 11.1, (C) 22.2 and (D) 33.3 MBq ^125^I seed irradiation. Cell cycle distribution was determined using flow cytometry with propidium iodide staining. (E) Quantitation of the cell cycle distribution (G0/G1, S and G2/M phases) of fibroblast following irradiation treatment. Values are presented as the mean ± standard deviation. ^*^P<0.05 vs. 0 MBq.

Apoptosis and cell cycle distribution in fibroblasts were evaluated using flow cytometry following exposure to ^125^I seed irradiation. As shown in Fig. 4, the apoptotic rate gradually increased in a dose-dependent manner with the activity of ^125^I seeds, with apoptotic rates of 6.73±0.57, 13.11±1.39 and 15.23±0.90% at 11.1, 22.2, and 33.3 MBq, respectively (P<0.05 vs*.* 0MBq). PI staining was used to determine cell cycle distribution, the results of which showed that increased ^125^I seed activity resulted in decreased S-phase populations and increased G1/G0-phase populations. However, G2/M populations were not significantly different among groups (Fig. 5).

### Safety and efficacy of a novel esophageal stent loaded with ^125^I seeds for prevention of benign restenosis in dogs

**Figure 6:**
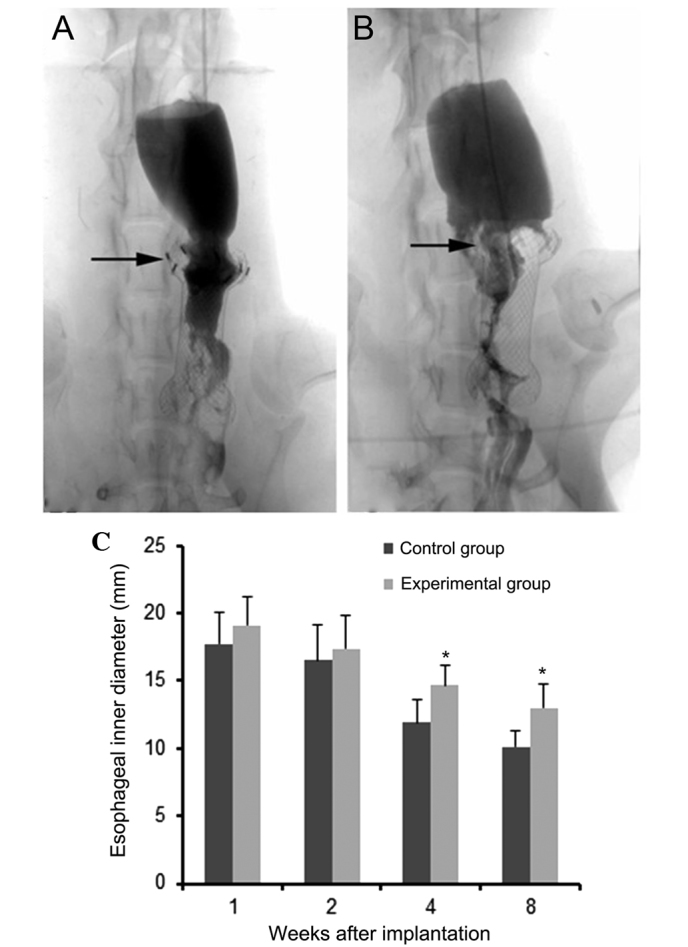
Digital subtracted angiography imaging following ^125^I seed esophageal stent implantation. Representative images for (A) an experimental animal with a ^125^I seed esophageal stent and (B) a control animal with an empty seed esophageal stent implantation at 8 weeks following implantation. Benign restenosis in the upper edge of the stent (shown by arrows) occurred in both groups. (C) Quantification of the inner diameters of the upper edge of stents. Values are presented as the mean ± standard deviation. ^*^P<0.05 vs. control group.

All stents were successfully released at the targeted sites. Following stent implantation, no stent migration, seed shedding or displacement were observed using DSA monitoring. At 1 and 2 weeks following implantation, a contrast agent was passed through the stents in the experimental and control groups; the agent passed smoothly with no significant stricture observed in the upper edge of stents. At four weeks, the contrast agent passed less smoothly and different levels of stricture were observed in the upper edge of the stents between experimental and control groups (data not shown). At eight weeks, the contrast agent passed with great difficulty due to significant stricture in both groups (Fig. 6). During DSA monitoring, no signs of contrast extravasation or esophageal perforation were observed in the experimental animals (Fig. 6A). Stricture was more pronounced in the control animals (Fig. 6B). In addition, no esophageal ulcerations or perforations were observed within the range of ^125^I seed irradiations on the upper edge following sacrification. Comparisons of intra-esophageal diameters at the upper edge of the stents in each group were measured at 1, 2, 4 and 8 weeks following implantation (Fig. 6C). These results showed that the intra-esophageal diameters gradually decreased in a time-dependent manner in each group. However, intra-esophageal diameters in the experimental group were significantly higher at 4 and 8 weeks compared with those in the control group (P<0.05), whereas at 1 and 2 weeks, there was no significant difference between groups (P>0.05).

**Figure 7:**
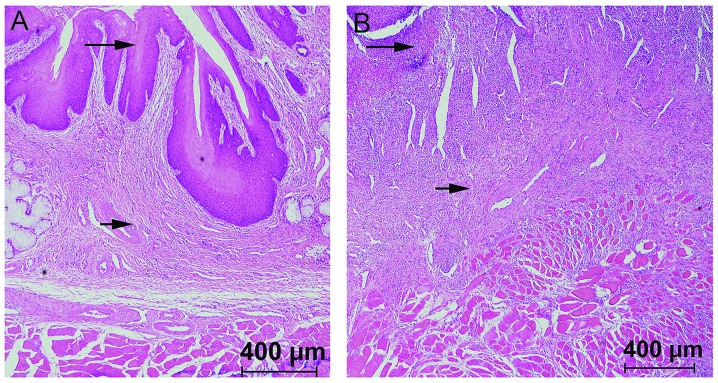
Hematoxylin-eosin staining at 8 weeks following ^125^I seed esophageal stent implantation. Representative sections from (A) experimental animals with ^125^I seed esophageal stent and (B) control animals with an empty seed esophageal stent (scale bar, 400 μm). In the control group, the mucosal layer of the esophageal tissue was no longer present (long arrows); in addition, extensive proliferation and fibrosis were observed in submucosal and muscular layers (short arrows).

Following sacrification, the stented esophageal segments were harvested along with the adjacent tissues for optical microscopy and immunohistochemical analyses (Fig. 7A and B). Using HE staining and microscopy, significant proliferation and thickening of the esophageal squamous tissue was observed in each group. Submucosal congestion, edema, vascular proliferation, infiltration of inflammatory cells as well as the formation of fibrous connective tissues and thickened esophageal muscle layers were also detected.

**Figure 8:**
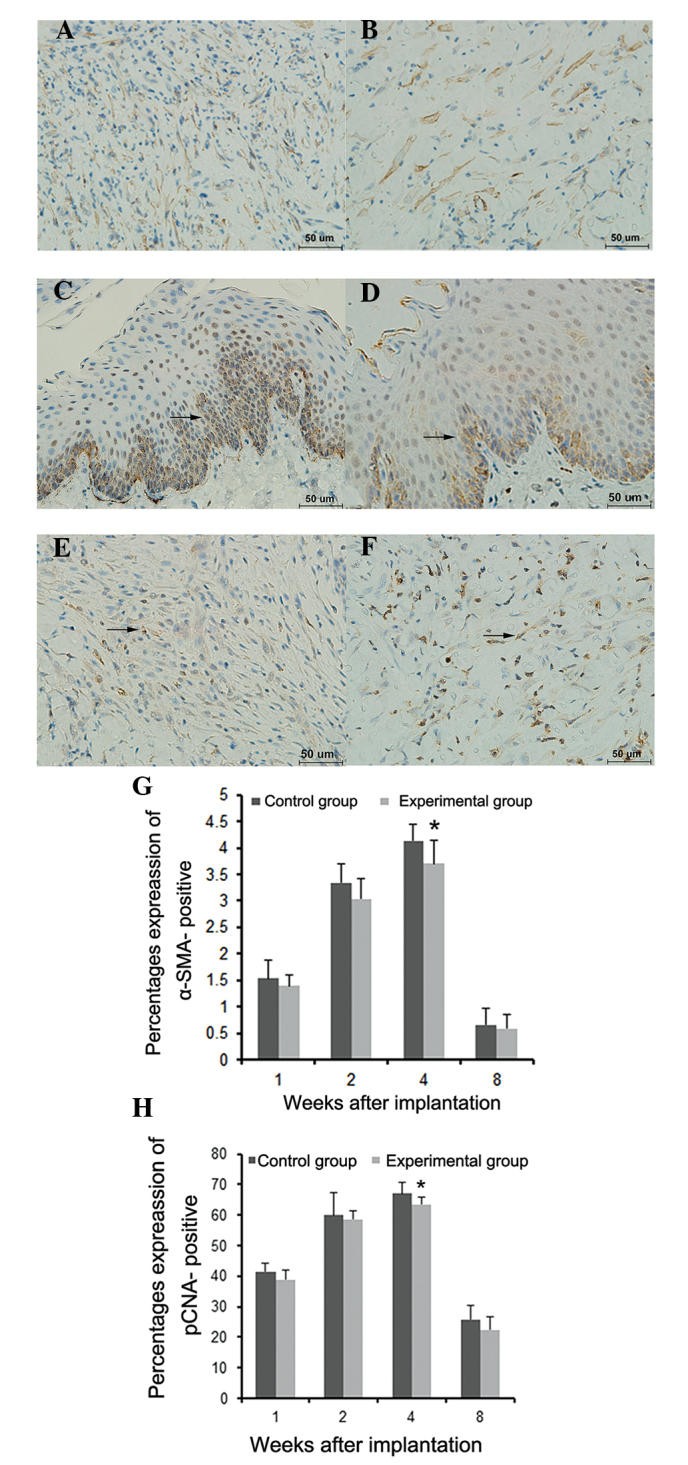
Immunohistochemical analysis of α-SMA and PCNA expressions in the esophageal submucosal layer following iodine-125 seeds esophageal stent implantation. (A) α-SMA and (C and E) PCNA expression in the control group at 4 weeks following surgery. (B) α-SMA and (D and F) PCNA expression in the experimental group at 4 weeks following surgery. Blue staining indicates nuclei positive for hematoxylin, while brown staining indicates expression of α-SMA and PCNA (arrows; scale bar, 50 μm). Histograms displaying the percentages of (G) α-SMA-positive and (H) PCNA-positive stained cells, as determined using optical density values. Values are presented as the mean ± standard deviation (n=4 each time point). ^*^P<0.05 vs*.* the control group. α-SMA, α-smooth muscle actin; PCNA, proliferating cell nuclear antigen.

Immunohistochemical data showed no obvious expression of α-SMA at either 1 or 8 weeks following implantation; however, α-SMA was detected in the submucosal layer at 2 weeks following implantation, which mainly consisted of fibroblast cytoplasmic, nuclear and neovascular staining. In addition, α-SMA expression was significantly lower in the experimental group at four weeks after implantation compared with that in the control group (P<0.05) (Fig. 8A, B and G).

Furthermore, immunohistochemical analysis showed PCNA expression 1 week following implantation. At 2 weeks post implantation, PCNA was primarily expressed in the basement membrane of the squamous epithelium and partially in the submucosal layer. In addition, PCNA expression in the experimental group was significantly lower at 4 weeks following implantation compared with that of the control group (P<0.05) (Fig. 8C–F and H).

As shown in Table II, the hydroxyproline contents of esophageal tissues at the upper edge of stents significantly increased in each group in a time-dependent manner. However, the hydroxyproline content in the experimental group was significantly lower compared with that in the control group at 4 and 8 weeks following implantation (P<0.05).

## Discussion

The *in vitro* irradiation model used in the present study has been widely used for ^226^Ra short distance irradiation treatment and radiation dosimetry, which was first described by Meredith (29)*.* This model was improved by Aird *et al* (27) for application in the study of ^125^I seed irradiation. An important concept, D/h, was implemented in this work, where D was the diameter of the irradiated circumference and h was the height between the irradiated circumference and the Petri dish. When D/h≤3, radiation sources only require to be placed at the circumference; however, when 3<D/h≤6, an additional 5% radiation source is required at the circumference, and when D/h>6, the radiation source requires to be placed in the coaxial circumferences as well as their centers. The purpose of this design was to obtain the best homogeneous dose distribution throughout Petri dishes. In the present study, 35-mm diameter petri dishes were used with small doses of ^125^I seeds, which had a very small effective radius; therefore, h=6 mm was selected. D/h≈5.8 was designed with eight ^125^I seeds equidistantly placed around the circumferences and one seed in the center to obtain a homogeneous ray; therefore, the variation of rays passing through the dishes was <10%. The average absorbed doses in the three experimental groups of ^125^I seed activity of 11.1, 22.2 and 33.3 MBq were 1.04, 2.08 and 3.12 cGy/h, respectively. The average absorbed doses in cells within 72 h were 0.75, 1.50 and 2.25 Gy, respectively. These data were determined using a software provided by Beijing Tianhangkelin Technology Development Co, Ltd (Radioactive seed source implantation treatment planning system; China State Food and Drug Administration, approval no. 3700398 of 2009).

The *in vitro* effects of ^125^I seed irradiation on fibroblasts were determined through the evaluation of cell proliferation, apoptosis and cell cycle distribution. The results of the present study demonstrated the significant inhibitory effects of ^125^I seeds on fibroblast proliferation in a dose-dependent manner. The highest ^125^I seed activity of 33.3 MBq showed the most potent IR of 45.33±2.59%. The effects of ^125^I seeds on apoptosis and cell cycle distribution were detected using Annexin V/PI double staining and PI staining, respectively; the results showed that apoptosis increased with ^125^I seed activity. In gastric cancer MKN45 cell lines, apoptosis rates of 13.67±1.58% and 14.00±1.87% were observed following irradiation with ^125^I seeds at 33.3 and 22.2 MBq, respectively, showing no statistically significant differences among groups (30). In addition, CL187 cells were treated with a similar *in vitro* ^125^I seed irradiation model and an apoptotic rate of 13.74±1.63% was reported in the group with an absorbed dose of 2 Gy (22). In the present study, cell cycle experiments demonstrated that ^125^I seed irradiation resulted in a dose-dependent increase of cells in G0/G1 phase, while S-phase populations decreased. G2 phase cell populations were not significantly different among groups, and no sub-G1 peaks were observed in any experimental group; by contrast, previous studies have reported a gradual dose-dependent elevation and the presence of sub-G1 peaks (22,23,31–33). The discrepancies between these data and the present findings may be due to the different doses as well as cell types that were studied; the previous studies mentioned the use of ^125^I seeds with higher activities. The low doses of ^125^I seeds used in the present study reflect those used clinically. It is likely that low doses of ^125^I seeds may result in blocking G1 to S-phase transition in fibroblasts, which results in the breakdown of cellular DNA into small fragments; therefore, no sub-G1 peaks were observed. In summary, all three activities of ^125^I seeds showed significant inhibitory effects on cell proliferation and certain pro-apoptotic effects in Beagle dog esophageal fibroblasts, with 33.3 MBp displaying the most potent effects. G1/S cell cycle arrest was induced in fibroblasts via irradiation of ^125^I seeds, resulting in the inhibition of fibroblast proliferation.

In the present study, a novel stent was developed through loading 8 ^125^I seeds with the same activity of 33.3 MBq onto the upper edge of a normal esophageal stent, which was then successfully implanted into the esophagus of Beagle dogs. No esophageal bleeding or perforation was observed using DSA monitoring and macroscopic observation 8 weeks following stent implantation, indicating the safety and feasibility of this technology. α-SMA is a known indicator of fibroblast proliferation and excessive extracellular matrix (ECM) secretion (34). During restricture, fibroblasts secrete large amounts of ECM, including different types of collagen, proteoglycans, elastin and certain adhesion proteins. Collagen proteins contain large amounts of hydroxyproline while most non-collagen proteins are hydroxyproline free (35); therefore, the level of fibroblast proliferation may be evaluated using hydroxyproline content and α-SMA expression assays. In the present study, α-SMA expression and hydroxyprolin content were found to be significantly decreased in the experimental group at four weeks following implantation compared with those of the control group. This therefore indicated that the proliferation of fibroblasts was effectively inhibited by ^125^I seeds. These findings were in accordance with the results of the intra-esophagus diameter experiments in the present study, which demonstrated that at 4 weeks following implantation, there were significant differences between the intra-esophagus diameters of the experimental and control groups. In addition, at 8 weeks following implantation, hyperplasia in the upper edge of the stent was significantly reduced in the experimental group compared with that in the control group.

In conclusion, the *in vitro* experiments showed that ^125^I seeds significantly inhibited cell proliferation via cell cycle arrest and apoptosis in Beagle dog esophageal fibroblasts. In addition, animal studies demonstrated that the application of a novel esophageal stent loaded with ^125^I seeds inhibited benign hyperplasia in the upper edge of the stent and, to a certain extent, relieving benign restenosis; this stent was also found to safely prevent benign restenosis following stent implantation.

## Figures and Tables

**Table I:** Effects of ^125^I seed irradiation on esophageal fibroblast proliferation.

Group	OD_490_	IR (%)
0 MBq	0.964±0.052	0
11.1 MBq	0.706±0.019^a^	26.81±1.96
22.2 MBq	0.632±0.031^a^	34.52±3.21
33.3 MBq	0.527±0.025^a^	45.33±2.59

IR was calculated according to the formula described above. Values are presented as the mean ± standard deviation.

a P<0.05 vs. 0 MBq.

IR, inhibition rate; OD_490_, optical density at 490 nm.

**Table II:** Hydroxyproline content in esophageal tissues in upper edge of stents following ^125^I seed esophageal stent implantation.

	Hydroxyproline (mg/l)	
	
Time period	Control group	Experimental group
1 week post surgery	55.21±2.36	51.85±1.53
2 weeks post surgery	67.91±1.94	64.88±2.29
4 weeks post surgery	90.59±1.98	80.56±1.97^a^
8 weeks post surgery	93.19±1.55	84.50±2.53^a^

Values are presented as the mean ± standard deviation (n=4 per time-point).

a P<0.05 vs. the control group.
